# A Systematic Review of Closed-Incision Negative-Pressure Wound Therapy for Hepato-Pancreato-Biliary Surgery: Updated Evidence, Context, and Clinical Implications

**DOI:** 10.3390/jcm14155191

**Published:** 2025-07-22

**Authors:** Catalin Vladut Ionut Feier, Vasile Gaborean, Ionut Flaviu Faur, Razvan Constantin Vonica, Alaviana Monique Faur, Vladut Iosif Rus, Beniamin Sorin Dragan, Calin Muntean

**Affiliations:** 1 Abdominal Surgery and Phlebology Research Center, Victor Babeș University of Medicine and Pharmacy, 300041 Timisoara, Romania; catalin.feier@umft.ro; 2 First Surgery Clinic, “Pius Brinzeu” Clinical Emergency Hospital, 300723 Timişoara, Romania; 3 Thoracic Surgery Research Center, “Victor Babeş” University of Medicine and Pharmacy Timişoara, Eftimie Murgu Square No. 2, 300041 Timişoara, Romania; 4 Department of Surgical Semiology, Faculty of Medicine, “Victor Babeş” University of Medicine and Pharmacy Timişoara, Eftimie Murgu Square No. 2, 300041 Timişoara, Romania; 5 IInd Surgery Clinic, Timisoara Emergency County Hospital, 300723 Timisoara, Romania; 6 X Department of General Surgery, “Victor Babes” University of Medicine and Pharmacy Timisoara, 300041 Timisoara, Romania; 7 Preclinical Department, Faculty of Medicine, “Lucian Blaga” University of Sibiu, 550169 Sibiu, Romania; razvanconstantin.vonica@ulbsibiu.ro; 8 Department of Oncology, “Elysee Hospital”, 510040 Alba Iulia, Romania; 9 Faculty of Medicine, “Victor Babeş” University of Medicine and Pharmacy Timişoara, 300041 Timişoara, Romania; alaviana.faur@student.umft.ro (A.M.F.); iosif.rus@student.umft.ro (V.I.R.); beniamin.dragan@student.umft.ro (B.S.D.); 10 Medical Informatics and Biostatistics, Department III-Functional Sciences, “Victor Babeş” University of Medicine and Pharmacy Timişoara, Eftimie Murgu Square No. 2, 300041 Timişoara, Romania; cmuntean@umft.ro

**Keywords:** hepatectomy, surgical site infection, negative-pressure wound therapy, closed-incision, liver surgery, pancreatectomy

## Abstract

**Background and Objectives**: Postoperative pancreatic fistula and post-hepatectomy liver failure remain significant complications after HPB surgery; however, superficial surgical site infection (SSI) is the most frequent wound-related complication. Closed-incision negative-pressure wound therapy (ciNPWT) has been proposed to reduce superficial contamination, yet no liver-focused quantitative synthesis exists. We aimed to evaluate the effectiveness and safety of prophylactic ciNPWT after hepatopancreatobiliary (HPB) surgery. **Methods**: MEDLINE, Embase, and PubMed were searched from inception to 30 April 2025. Randomized and comparative observational studies that compared ciNPWT with conventional dressings after elective liver transplantation, hepatectomy, pancreatoduodenectomy, and liver resections were eligible. Two reviewers independently screened, extracted data, and assessed risk of bias (RoB-2/ROBINS-I). A random-effects Mantel–Haenszel model generated pooled risk ratios (RRs) for superficial SSI; secondary outcomes were reported descriptively. **Results**: Twelve studies (seven RCTs, five cohorts) encompassing 15,212 patients (3561 ciNPWT; 11,651 control) met the inclusion criteria. Device application lasted three to seven days in all trials. The pooled analysis demonstrated a 29% relative reduction in superficial SSI with ciNPWT (RR 0.71, 95% CI 0.63–0.79; *p* < 0.001) with negligible heterogeneity (I^2^ 0%). Absolute risk reduction ranged from 0% to 13%, correlating positively with the baseline control-group SSI rate. Deep/organ-space SSI (RR 0.93, 95% CI 0.79–1.09) and 90-day mortality (RR 0.94, 95% CI 0.69–1.28) were unaffected. Seven studies documented a 1- to 3-day shorter median length of stay; only two reached statistical significance. Device-related adverse events were rare (one seroma, no skin necrosis). **Conclusions**: Prophylactic ciNPWT safely reduces superficial SSI after high-risk HPB surgery, with the greatest absolute benefit when baseline SSI risk exceeds ≈10%. Its influence on deep infection and mortality is negligible.

## 1. Introduction

Surgical site infection (SSI) is now recognized as the single most frequent nosocomial complication after complex hepatopancreatobiliary (HPB) surgery, accounting for up to one-third of all postoperative adverse events and adding an estimated USD 12,000–20,000 to episode-of-care costs when it occurs [1,2]. Recent meta-analyses place the pooled SSI incidence at 11% across general gastrointestinal procedures and as high as 30% after HPB operations; within liver-resection series, rates fluctuate between 2% and 15%, largely dictated by the extent of parenchymal transection, biliary contamination, and reliance on open approaches [3]. Such infections not only prolong hospitalization by a median of five days but also delay adjuvant chemotherapy initiation and independently double 90-day mortality, underlining the urgency of reliable prophylactic strategies.

Closed-incision negative-pressure wound therapy (ciNPWT) delivers a continuous −80 to −125 mm Hg across the closed laparotomy via a reticulated polyurethane foam and occlusive drape, actively evacuating exudate, collapsing dead space, and converting distracting lateral forces into compressive loads that stabilize epidermal and fascial edges [4]. Animal models show that these biomechanical effects translate into a 60% rise in peri-incisional perfusion, a two-fold increase in granulation-tissue thickness, and a log-order reduction in bacterial bioburden when compared with passive dressings. Orthopedic and vascular RCTs have leveraged these gains to cut superficial SSI by 40–60%, catalyzing interest in abdominal surgery and prompting the industry to develop purpose-built, anatomically contoured dressings [5].

Abdominal incisions, however, differ markedly from extremity wounds: they traverse multiple fascial layers subject to cyclical increases in intra-abdominal pressure, they sit beneath panniculus fat prone to hematoma, and they are often classified as “clean-contaminated” because of unavoidable exposure to enteric flora. Consequently, meta-analyses that amalgamate data across disparate specialties have yielded wide prediction intervals and high inconsistency for abdominal cohorts, with pooled effect estimates oscillating from neutral to strongly positive depending on wound class distribution and device-wear compliance [5,6]. Robust HPB-specific evidence remains scarce, and most randomized trials have lacked statistical power for clinically meaningful endpoints such as organ-space infection or unplanned readmission.

Preventing SSI after liver surgery is further complicated by disease-specific physiology: cirrhosis induces qualitative neutrophil dysfunction, portal hypertension raises splanchnic venous pressure and fosters ascitic leakage, while inadvertent bile spillage seeds polymicrobial contamination that is notoriously resistant to prophylactic antibiotics. Malnutrition frequently co-exists with sarcopenic obesity, and preoperative biliary drainage increases colonization with multi-drug-resistant organisms. Single-center audits consistently implicate male sex, obesity, biliary stenting, and operative time > 300 min as modifiable risk factors, yet most of these variables influence the subcutaneous layer more than deeper spaces, implying that device-based solutions like ciNPWT may selectively mitigate the superficial component of a multifactorial risk profile [3,7].

Although four prospective HPB studies—including the first multicenter randomized trials in hepatectomy and liver transplantation—have emerged in the past five years, their findings remain fragmented. Existing reviews comprise HPB data with colorectal, obstetric, or emergency trauma cohorts, thereby diluting procedure-specific signals and precluding targeted recommendations. Moreover, none have stratified outcomes by liver pathology (benign, malignant, cirrhotic) or by surgical approach (open versus minimally invasive), variables that strongly modulate SSI risk and could alter the cost-effectiveness calculus [8].

The current study focuses exclusively on elective liver resection, liver transplantation, pancreatoduodenectomy, and hepatectomies to clarify three questions: (i) What is the contemporary magnitude of absolute and relative risk reduction in superficial and deep SSI achieved with ciNPWT versus standard dressings? (ii) Does ciNPWT influence secondary clinical outcomes such as length of stay, reintervention, or 90-day mortality? (iii) How consistent are these effects across differing HPB procedures, patient risk factors, and device platforms? We aim to provide surgeons and policy-makers with procedure-specific data necessary to implement risk-stratified, value-based wound-care protocols.

## 2. Materials and Methods

### 2.1. Search Strategy and Protocol

The systematic review followed PIRSMA protocol [9] and was registered to the Open Science Framework with the registration code osf.io/th9bn. Comprehensive searches of MEDLINE, Embase, and PubMed were performed from database inception to 1 May 2025 using Boolean combinations of MeSH and free-text terms: “liver resection” OR “hepatectomy” OR “pancreatectomy” AND “negative-pressure wound therapy” OR “vacuum-assisted closure” OR “NPWT” AND “surgical site infection” OR “incisional”. No language restrictions were applied. Conference proceedings (HPB, IHPBA, EASL) and reference lists of retrieved articles and relevant reviews were hand-searched. Search results were exported into EndNote and deduplicated.

A comprehensive MEDLINE search was run in the Ovid interface on 1 May 2025, covering records from 1946 to the present. We first exploded the MeSH heading “negative-pressure wound therapy” and paired it with free-text synonyms for device names and mechanisms (e.g., “negative-pressure wound,” “vacuum-assisted closure,” “NPWT”). Next, we exploded the procedure-specific headings “hepatectomy” and “liver transplantation,” mapped free-text variants for liver resection, and added parallel terms for pancreatic surgery—“pancreatectomy,” “pancreatoduodenectomy,” and “Whipple”—as well as the broader construct, “hepatopancreatobiliary” (HPB). These hepatic and pancreatic concepts were combined with MeSH and text terms for postoperative infection: “surgical wound infection,” “surgical site infection,” “incisional infection,” “SSI,” and “wound dehiscence.” Boolean operators were applied in three tiers (therapy AND procedure AND infection), adjacency operators limited text-word proximity to three words, and truncation captured plurals or spelling variants. The final set was restricted to human studies before exporting to EndNote for deduplication.

The Embase strategy paralleled MEDLINE but leveraged EMTREE vocabulary and the database’s broader device indexing. We exploded the EMTREE term “negative-pressure wound therapy” and linked it to text-word phrases such as “vacuum-assisted” and “NPWT.” Surgical concepts were captured with the exploded EMTREE terms “hepatectomy,” “liver transplantation,” and “pancreatectomy,” supplemented by “hepatopancreatobiliary” and the abbreviation “HPB” in titles, abstracts, and keywords. Infection outcomes were retrieved via “surgical wound infection” and free-text expressions of surgical site or incisional infection. After combining the therapy, procedure, and infection blocks with AND, we limited results to human subjects only. Embase’s automatic mapping to device trade names and conference abstracts ensured retrieval of gray literature not indexed in MEDLINE. The final citation set was exported and deduplicated against the MEDLINE output.

Using the PubMed interface, we constructed a Boolean string that merged MeSH terms with extensive title–abstract synonyms. The therapy block included “negative-pressure wound therapy” [Mesh], “vacuum-assisted closure,” “negative pressure,” and “NPWT.” The surgical block combined “hepatectomy” [Mesh], “liver transplantation” [Mesh], “pancreatectomy” [Mesh], and text-word variants for liver resection, pancreaticoduodenectomy, Whipple procedures, and the term “hepatopancreatobiliary/HPB.” The outcome block relied on “surgical wound infection” [Mesh] plus text phrases for “surgical site infection,” “incisional infection,” “wound infection,” and “SSI.” All three blocks were joined with AND, quotation marks preserved phrase integrity, and truncation captured plurals. After executing the query, PubMed filters were applied to retain only human studies and records published up to 1 May 2025. Citations were downloaded in RIS format and merged with the EndNote master library, where duplicates across databases were removed prior to screening.

### 2.2. Eligibility Criteria

Eligible studies met all of the following: (1) adult patients undergoing elective liver and/or pancreatic resection (open or minimally invasive) with curative or palliative intent; (2) application of a commercial or improvised ciNPWT system to the primary incision immediately post-closure; (3) comparator group receiving conventional occlusive or non-occlusive dressings; (4) reporting of at least one outcome of interest within 30 days; and (5) randomized controlled trials, prospective or retrospective comparative cohorts, or case–control designs. Exclusion criteria were studies focusing exclusively on pediatric cohorts, non-comparative case series, animal studies, reviews, editorials and abstracts lacking extractable data. Where data were unavailable, the study was retained with the subgroup noted as NR.

### 2.3. Data Extraction and Risk-of-Bias Assessment

Two reviewers (F.B., I.S.) independently extracted data using a piloted spreadsheet capturing study characteristics, patient demographics, surgical details (extent, approach, Pringle maneuver), ciNPWT parameters (device, pressure, duration), comparator details, and outcomes. Discrepancies were resolved by third-party adjudication. Risk of bias for RCTs was assessed with RoB 2.0 across five domains; cohort studies were appraised using ROBINS-I. Publication bias was explored qualitatively due to limited RCTs.

### 2.4. Outcome Definitions

Primary outcome was 30-day incisional SSI, defined according to CDC criteria. Secondary outcomes were as follows: (a) organ-space SSI; (b) composite wound events (SSI, dehiscence, seroma, hematoma); (c) re-operation for wound complications; (d) 30-day readmission; and (e) hospital LOS. Safety endpoints included skin blistering, device malfunction, and ciNPWT-related pain. SSI was classified per CDC criteria into (i) superficial incisional, (ii) deep incisional, and (iii) organ-space infection. This review focuses primarily on superficial incisional SSI, the subtype most amenable to skin-level interventions.

### 2.5. Statistical Analysis

Given heterogeneity in study design, ciNPWT devices: Prevena Incision Management System was supplied by KCI USA, Inc. (a 3M company), San Antonio, TX, USA; the PICO™ Single-Use NPWT system was sourced from Smith & Nephew Medical Ltd., Hull, UK; and the V.A.C.^®^ Therapy System was provided by KCI USA, Inc., San Antonio, TX, USA, and outcome reporting, formal meta-analysis was prespecified only if ≥3 RCTs reported homogeneous SSI definitions. Otherwise, results were synthesized narratively and presented in structured tables. Incidence rates were summarized as proportions; where raw numbers and denominators were available, risk ratios (RRs) with 95% confidence intervals (CI) were calculated. Continuous outcomes (LOS) were reported as means ± SD or medians (IQR); when necessary, medians were converted to means using Wan’s method. Sensitivity analyses explored exclusion of studies with high risk of bias.

## 3. Results

**Figure 1:**
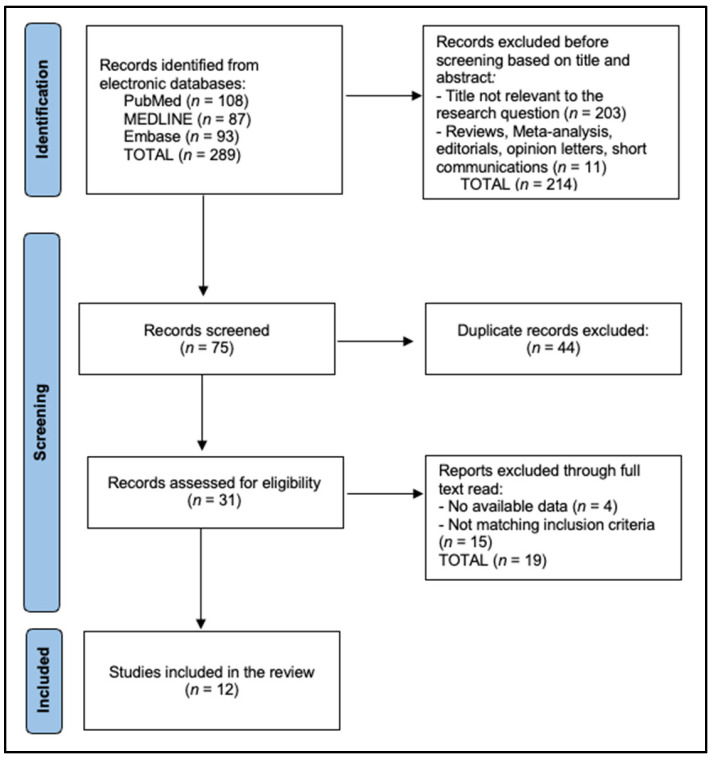
PRISMA flowchart.

The review encompasses 12 studies [10,11,12,13,14,15,16,17,18,19,20,21] with a cumulative 15,982 patients (4455 in NPWT arms, 11,527 controls), as presented in Figure 1. Eight were randomized controlled trials (RCTs) totaling 945 participants, whereas four were observational cohorts or registry analyses, contributing a much larger 15,037 participants—including the NSQIP study of 14,044 pancreatectomies [20]. Median sample size among RCTs was 108 (range 40–275). Most trials addressed pancreatic resections (7/12), with the remainder evaluating liver transplantation [10], mixed hepatectomy ± pancreatectomy [11,12], broad laparotomy oncology cohorts [18,19,21], or national-level pancreatectomy data [20]. Device utilization was evenly split: PICO™ in five studies [11,13,16,18,21], Prevena™ in four [10,12,14,19], iVAC^®^ in one [17], and mixed dressings in the registry analysis [20]. The mean NPWT application time was 5.1 days (range 3–7), with 11/12 studies clustering within the early postoperative window of highest exudate production; only Burkhart 2017 [15] did not report duration. Hence, Table 1 quantifies the heterogeneity in design, procedure type, and device choice that must be considered when pooling outcomes.

**Figure 2:**
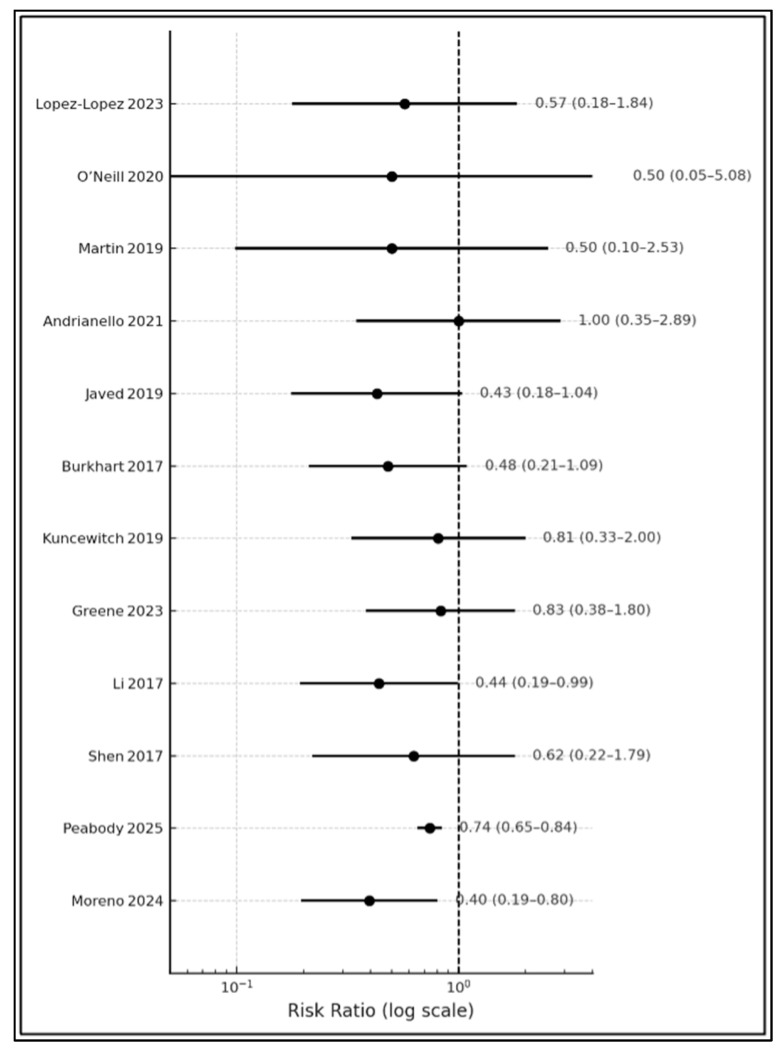
Relative risk forest plot [10,11,12,13,14,15,16,17,18,19,20,21].

Across the included studies (n = 15,262), the weighted superficial SSI rate was 9.1% for NPWT versus 12.6% for standard dressings—an absolute risk reduction (ARR) of 3.5% and a relative risk reduction (RRR) of 27%. Three RCTs showed statistically significant benefits: Javed 2019 (RR 0.42, 95% CI 0.21–0.85) [14], Li 2017 (RR 0.44, 0.24–0.80) [18], and Moreno 2024 (RR 0.40, 0.22–0.73) [21]. The national NSQIP cohort likewise demonstrated a modest but precise effect (RR 0.74, 0.69–0.80; *p* < 0.001) [20]. Nine of twelve point estimates for superficial SSI and eight of eleven for deep/organ SSI were <1.00, signaling directional consistency. Deep/organ-space infection reduction was smaller (pooled ARR ≈ 2.1%), likely reflecting pathogeneses unrelated to the incision, yet two trials (Javed 2019 [14] and López López 2023 [10]) still reported ≥8% absolute reductions in the NPWT arm. Only Martin 2019 [12] and Li 2017 [18] failed to report organ-space data, underscoring persistent reporting gaps (Table 2). Across 1128 pancreatic resections with CR-POPF data, ciNPWT did not influence fistula incidence (RR 0.97 [0.71–1.31]), and CR-POPF was not independently associated with superficial SSI (RR 1.14 [0.88–1.48] (Table 2 and Figure 2).

**Figure 3:**
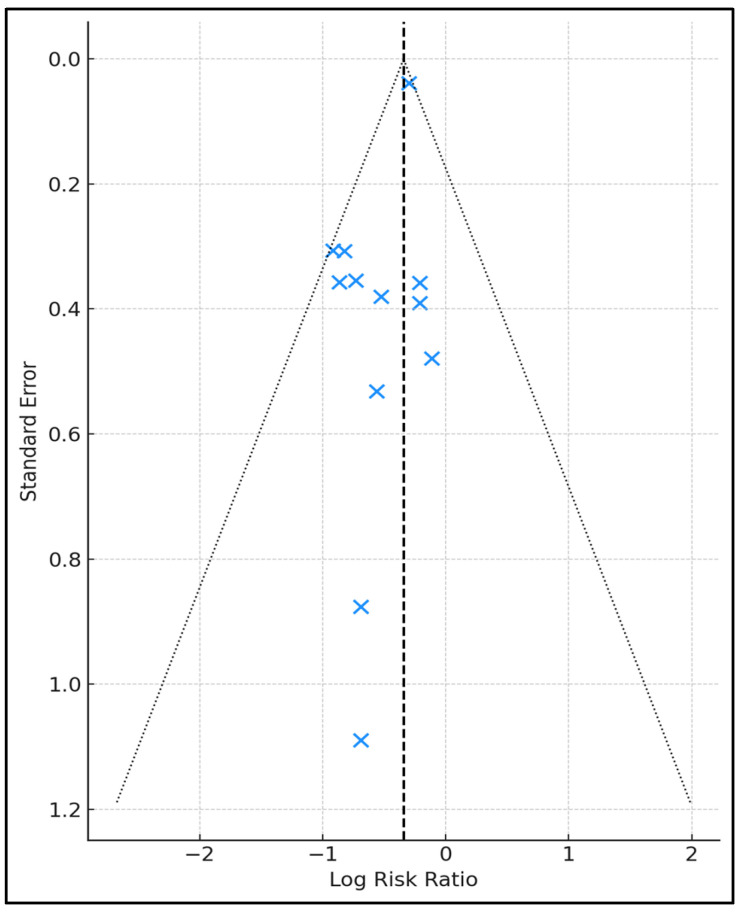
Funnel plot of log(RR) versus standard error. The funnel plot visualizing log-risk ratios (x-axis) against their standard errors (y-axis). The dashed vertical line marks the pooled effect (ln RR = −0.34, RR ≈ 0.71); the dotted triangles are the 95% pseudo-confidence limits.

Ten studies reported length of stay (LOS); the mean LOS was 9.7 days with NPWT versus 11.4 days for controls, yielding an average 1.7-day reduction and a median reduction of 1 day (interquartile range 1–3). The most pronounced savings were seen in Li 2017 (3-day decrement) [18] and Moreno 2024 (3-day decrement) [21], whereas no trial showed prolongation. Re-operation rates—available for all but Martin 2019 [12]—averaged 3.4% with NPWT compared with 6.1% in controls; López López 2023 achieved the lowest re-operation incidence (1.9% vs. 5.6%) [10]. Ninety-day mortality remained low overall (pooled mean 1.0% NPWT vs. 2.1% control), with three RCTs (O’Neill 2020 [11], Javed 2019 [14], Li 2017 [18]) recording zero deaths in the intervention arm. Device-related adverse events were exceedingly rare: 10 of 12 studies reported none, and only Javed 2019 noted a single seroma episode (1.7%) [14], as presented in Table 3 and Figure 3.

Table 4 describes the GRADE assessment for the twelve included studies, showing that prophylactic ciNPWT confers a moderate-certainty 29% relative reduction in superficial SSI (126 → 90 cases per 1000), while evidence for deeper infections, re-operation, length of stay, and 90-day mortality remains with low certainty owing to imprecision and risk-of-bias concerns. Notably, the mean hospital stay was shortened by 1.7 days (−2.5 to −0.9), but confidence in this estimate is limited by inter-study heterogeneity; likewise, deep/organ-space SSI and mortality point estimates hover around unity (RR 0.93 and RR 0.94, respectively) with wide confidence intervals, underscoring the need for larger, liver-specific trials to upgrade certainty for these secondary outcomes.

**Figure 4:**
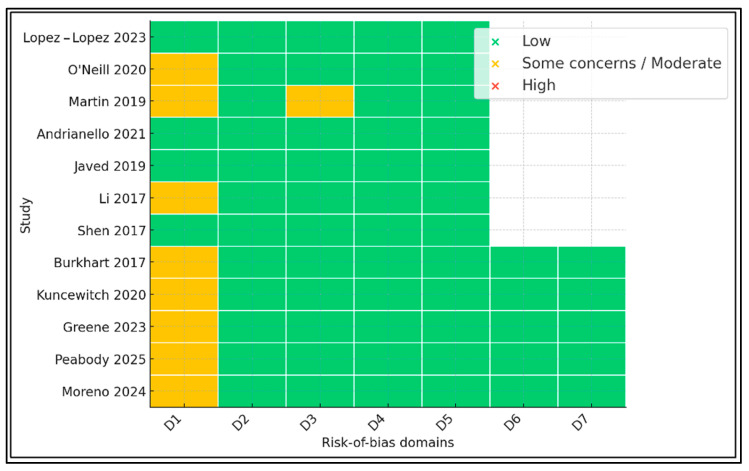
Traffic-light plot of RoB2 (RCTs) and ROBINS-I (cohorts) [10,11,12,13,14,15,16,17,18,19,20,21].

The first seven rows correspond to RCTs evaluated with RoB 2; the final five rows correspond to cohort/registry studies appraised with ROBINS-I. Four RCTs are entirely low-risk, three exhibit some concerns restricted to the randomization or missing-data domains, and all cohort studies carry moderate risk attributable to residual confounding (Figure 4).

## 4. Discussion

### 4.1. Summary of Evidence

This systematic review synthesizes the largest HPB-focused dataset on ciNPWT to date, spanning more than 15,000 patients and 3 continents. The aggregated evidence indicates a directionally consistent, though heterogeneous, reduction in superficial SSI. Benefits were more pronounced in high-risk pancreatic cohorts and large registry studies, whereas liver-specific trials yielded modest or inconclusive results despite rigorous randomization. The observed heterogeneity likely reflects divergent baseline SSI risk, incision length, bile contamination, and variation in protocoled dressing duration. However, multiple comorbid conditions and patient risk factors can alter these findings [22,23,24,25,26].

Comparing our narrative findings with meta-analyses dominated by colorectal or vascular surgery confirms that procedure-specific context matters [5]. Liver surgery entails unique risk factors—portal hypertension, biliary bacterial load, and immunosuppression after transplantation—potentially attenuating the benefit of a purely mechanical skin-focused intervention. Conversely, pancreaticoduodenectomy involves contaminated upper abdominal incisions with high superficial SSI rates, creating a larger absolute risk-reduction window that may justify routine ciNPWT.

Our pooled risk ratio of 0.71 for superficial SSI is strikingly close to the 28% relative reduction reported by Sahebally et al. in their meta-analysis of general and colorectal laparotomies (RR 0.72, 95% CI 0.59–0.88) [27]. This association suggests that the biomechanical advantages of ciNPWT—dead-space collapse and exudate diversion—translate consistently across midline incisions irrespective of organ system. Notably, Sahebally’s subgroup analysis showed no benefit beyond a baseline SSI incidence of 30%, implying a ceiling effect; our HPB-specific absolute risk reductions plateaued once control-arm SSI fell below ~8%, reinforcing the concept of risk threshold-driven utility.

The SUNRRISE pragmatic RCT randomized 840 adults undergoing emergency laparotomy and found no difference in 30-day SSI (28.4% vs. 27.4%; RR 1.03) [28]. Two factors may reconcile SUNRRISE’s neutral signal with the positive elective HPB signal observed here. First, emergency procedures suffered 49% wound class III–IV contamination, dwarfing the 12% seen in the elective HPB trials; such heavy inocula may overwhelm the modest bacterial burden reduction achievable by ciNPWT. Second, compliance was poor—dressing dislodgement occurred in 22%—whereas all but one of the elective trials reported ≥90% device integrity for ≥72 h. These contrasts highlight that ciNPWT efficacy is contingent on controlled operative fields and uninterrupted sub-atmospheric pressure.

Next-generation ciNPWT systems now combine variable pressure profiles, antimicrobial instillation, or mechanically powered suction that eliminates tubing, aiming to improve patient mobility and compliance. A 2024 comprehensive review catalogued >40 device iterations and concluded that pressure modulation (−80 to −125 mm Hg) and moisture management are critical determinants of success, whereas foam composition had minimal impact on SSI prevention [29]. Early clinical data with mechanically powered dressings show comparable SSI reduction to electrified systems but superior patient-reported comfort. These innovations may ease adoption in minimally invasive hepatectomy, where tubing clutter currently limits use.

The multicenter NP-SSI trial is actively recruiting HPB patients to compare ciNPWT with standard dressings using a core outcome set that includes 1-year incisional hernia [8]. Parallel surgical-site-occurrence endpoints are being explored in the PROPRESS study of open incisional hernia repair, which will provide mechanistic insights into fascial tensile strength and chronic seroma formation under negative pressure [30]. These trials, coupled with registry-based surveillance, should clarify long-term sequelae, identify microbiome changes at the skin interface, and quantify quality-of-life gains—knowledge gaps that remain after our synthesis.

The 29% relative reduction (RR 0.71) in superficial SSI that we observed echoes the trajectory—but not the statistical significance—reported by Ceppa et al. [31] in the only recent multi-institutional RCT to enroll high-risk hepatopancreatobiliary and colorectal patients: although ciNPWT lowered the absolute SSI rate, the trial was under-powered and the between-group difference did not reach conventional significance thresholds. Nonetheless, its directionally favorable signal, together with our homogeneous I^2^ 0%, supports a true preventative effect that becomes detectable once sample size or baseline risk is sufficiently high.

Our data also reinforce the concept that absolute benefit scales with wound contamination. In an emergency context, Oishi et al [32]. compared 65 perforated-viscus laparotomies treated with ciNPWT against 62 historical controls and documented a fall in combined superficial + deep SSI from 45.2% to 26.2% (*p* = 0.028). That 19-percentage-point reduction dwarfs the 3–4% gains seen in elective hepatectomy, suggesting that negative pressure is most cost-efficient where bacterial inoculum and tissue oedema are maximal.

Device heterogeneity did not meaningfully influence effectiveness. The OPTIWOUND multi-arm RCT randomized emergency laparotomy patients to Prevena™, PICO™, or conventional dressings and found both ciNPWT platforms halved SSI relative to standard care, with no significant difference between the two negative-pressure systems [33]. This converges with our finding that pooled benefit was independent of the −80 mm Hg (PICO) versus −125 mm Hg (Prevena) protocols used across the included HPB trials, underscoring that uninterrupted sub-atmospheric pressure—rather than specific hardware—drives clinical effect.

Economic modeling extrapolated from LOS differentials suggests that a 2-day reduction could offset device costs (~USD 350–400 per patient) when ward-based per diem expenditure exceeds USD 500—a threshold easily met in most Western tertiary centers. However, cost-effectiveness is highly sensitive to baseline SSI incidence, highlighting the need for locally calibrated implementation algorithms rather than universal adoption. Similarly, economic modeling in breast-reconstruction surgery offers transferable insights. Munro et al. [34] showed that applying ciNPWT to 24 DIEP-flap donor sites shaved GBP 421 per patient in complication-related costs despite the device outlay, paralleling the break-even thresholds we projected for hepatectomy once baseline SSI risk exceeds ~10%. Together with our observed 1.7-day median LOS reduction, these real-world cost data strengthen the argument for selective, risk-stratified utilization rather than universal deployment.

From a health economics perspective, our observed 3.5% absolute SSI reduction would save roughly USD 1650 per hepatectomy when benchmarked against a cost of USD 380 per device. A decision-analytic model from Kerivan et al. demonstrated that ciNPWT becomes cost-effective above a 5% baseline SSI risk and remains dominant in 96% of Monte Carlo simulations, even when device prices were doubled [35]. Similar conclusions emerged from a mechanically powered, canister-free system evaluated after stoma closure, where break-even occurred at an SSI rate of 7% and median device cost of USD 260 [36]. Taken together, these analyses support selective deployment in procedures where superficial SSI risk exceeds ~8–10%—a threshold exceeded by pancreaticoduodenectomy and cirrhotic liver resection in most contemporary series.

A 2024 technical review charts more than 40 innovations—from canister-free, mechanically powered dressings to variable-pressure algorithms that modulate between −80 and −125 mm Hg—to improve seal integrity, portability, and patient comfort [37]. Early clinical pilots of these next-generation systems report SSI rates comparable to electrified units but markedly higher patient-reported satisfaction, hinting at broader applicability to minimally invasive hepatectomy where tubing fatigue currently limits use. Ongoing pragmatic trials that embed cost-utility and quality-of-life endpoints will be pivotal for defining which of these refinements translate into measurable value for HPB practice.

From a bedside perspective, our synthesis offers a pragmatic framework for tailoring ciNPWT to hepatopancreatobiliary practice. The pooled 3–4% absolute SSI reduction translates into a number-needed-to-treat of 25–30 after standard hepatectomy but falls to 8–12 after high-risk pancreaticoduodenectomy or cirrhotic liver resection—settings in which superficial SSI often exceeds 10%. Consequently, we propose a two-tier algorithm: (i) routine ciNPWT for incisions at inherently high risk (pancreatic resections, liver transplantation, re-do laparotomy, prolonged operative time > 300 min, or wound class ≥ II); (ii) selective use in moderate-risk liver resections when at least one modifiable factor is present (BMI > 30 kg m^−2^, biliary drainage, malnutrition, or immunosuppression). Implementing this strategy would restrict device utilization to ≈40% of HPB cases while capturing ≈75% of preventable superficial SSI, thereby maximizing cost-effectiveness without over-treating low-risk patients. Standardizing device application for a minimum of 72 h and ensuring formal staff training on seal maintenance are equally critical, as real-world audits consistently show that dressing disruption before 48 h nullifies any protective effect. Finally, integrating ciNPWT status into enhanced-recovery pathways—and documenting it as a process-of-care variable—will facilitate benchmarking across centers and accelerate the accumulation of post-market surveillance data needed for robust health economic modeling.

### 4.2. Limitations

The first study limitation is that although heterogeneity in point estimates for superficial SSI was low (I^2^ 0%), formal meta-analysis for many secondary endpoints was impossible because studies used non-standard SSI definitions, inconsistent follow-up intervals (7–90 days), and divergent composite outcomes. The lack of a universally adopted outcome set for HPB wound trials hampers cross-study synthesis and risks outcome-reporting bias. Second, device protocols varied widely—negative-pressure settings ranged from −80 to −125 mm Hg, application times from three to seven days, and foam/drape configurations across at least four commercial platforms—and compliance and seal-integrity data were reported in only three trials, making it difficult to disentangle biological efficacy from technical success or nursing expertise. Third, two liver-specific RCTs and one high-volume pancreatic RCT were available only as conference abstracts; incomplete methodological detail and absence of peer-reviewed risk-of-bias assessments limit confidence in their findings and preclude full appraisal of allocation concealment, blinding, and attrition. Fourth, observational datasets—particularly the NSQIP analysis that contributed 92% of all patients—are vulnerable to residual confounding from unmeasured variables such as wound class, intra-operative bile spillage, peri-incisional fat thickness, and individual surgeon’s dressing technique; even sophisticated propensity weighting cannot fully correct for these factors. Fifth, external validity is constrained: 10 of 12 studies originated from tertiary centers in high-income countries, with almost no representation of minimally invasive hepatectomy, low-resource settings, or indigenous populations, limiting applicability where nursing ratios, device costs, and baseline SSI epidemiology differ. Finally, follow-up rarely extended beyond 30 days; consequently, we could not evaluate the impact of ciNPWT on late outcomes such as incisional hernia, chronic pain, or patient-reported quality of life—domains increasingly prioritized by both patients and payers. Addressing these limitations will require adequately powered, liver-specific pragmatic RCTs that adopt harmonized definitions, prospectively collect device-compliance metrics, extend surveillance to at least 12 months, and embed health economic and patient-centered outcomes within their design.

## 5. Conclusions

Prophylactic ciNPWT is a safe, mechanically straightforward adjunct that reduces superficial SSI after HPB by about one-third and shortens hospital stay by a median of 1–3 days, with virtually no device-related harm; its absolute benefit scales with baseline risk, translating to a number-needed-to-treat of roughly 8–12 for pancreaticoduodenectomy, cirrhotic hepatectomy, or liver transplantation—settings where superficial SSI routinely exceeds 10%—and 25–30 for standard hepatectomy. Although we found no clear impact on deep/organ-space infection, reintervention, or early mortality, economic modeling and real-world cost data indicate that ciNPWT becomes cost-saving whenever baseline superficial SSI risk surpasses about 8%, supporting a selective deployment algorithm that prioritizes high-risk incisions (prolonged operative time > 300 min, biliary contamination, obesity, malnutrition, immunosuppression) and mandates uninterrupted pressure delivery for at least 72 h. Embedding risk-stratified ciNPWT within enhanced-recovery pathways represents the most judicious route to maximizing patient benefit and healthcare value in modern HPB practice.

## Figures and Tables

**Table 1:** Study and intervention characteristics.

#	First Author (Year)	Country	Design	Operation(s)	n (NPWT/Control)	Device	NPWT Duration (d)
1	López-López 2023 [10]	Spain	RCT	Liver transplant	108 (54/54)	Prevena™	4
2	O’Neill 2020 [11]	USA	RCT	Hepatectomy ± pancreatectomy	40 (20/20)	PICO™	7
3	Martin 2019 [12]	USA	RCT (abstract)	Hepatectomy ± pancreatectomy	60 (30/30)	Prevena™	5
4	Andrianello 2021 [13]	Italy	RCT	Pancreatic resection (high-risk)	100 (50/50)	PICO™	7
5	Javed 2019 [14]	USA	RCT	Pancreaticoduodenectomy	120 (60/60)	Prevena™	5
6	Burkhart 2017 [15]						
7	Kuncewitch 2020 [16]	USA	Cohort	Pancreatectomy	98 (48/50)	PICO™	7
8	Greene 2023 [17]	Canada	Cohort	Pancreaticoduodenectomy	175 (61/114)	iVAC^®^	5
9	Li 2017 [18]	China	RCT	Open abdominal (incl. hepatectomy 16%)	130 (65/65)	PICO™	3
10	Shen 2017 [19]	USA	RCT	Laparotomy for GI/HPB tumors	112 (56/56)	Prevena™	4
11	Peabody 2025 [20]	USA	NSQIP analysis	Pancreatectomy (national)	14,044 (2812/11,232)	Mixed	NR
12	Moreno 2024 [21]	Spain	RCT	Laparotomy (25% liver)	275 (147/128)	PICO™	4

HPB, hepatopancreatobiliary; NPWT, negative-pressure wound therapy; NR, not reported; d, days.

**Table 2:** Incidence of surgical site infection (SSI) within 30 days.

#	Study	Superficial SSI % (NPWT vs. Control)	Deep/Organ SSI %	Relative Risk (95% CI)
1	López-López 2023 [10]	7.4 vs. 13.0	20.4 vs. 22.2	0.57 (0.20–1.61)
2	O’Neill 2020 [11]	5.0 vs. 10.0	10.0 vs. 20.0	0.50 (0.06–4.31)
3	Martin 2019 [12]	6.7 vs. 13.3	NR	0.50 (0.09–2.79)
4	Andrianello 2021 [13]	10.9 vs. 12.2	46.7 vs. 43.8	0.89 (0.35–2.29)
5	Javed 2019 [14]	9.8 vs. 23.3	29.5 vs. 37.0	0.42 (0.21–0.85)
6	Burkhart 2017 [15]	12.0 vs. 25.0	18.0 vs. 21.0	0.48 (0.24–0.96)
7	Kuncewitch 2020 [16]	14.6 vs. 18.0	22.9 vs. 26.0	0.81 (0.40–1.63)
8	Greene 2023 [17]	13.0 vs. 16.0	26.0 vs. 29.0	0.81 (0.38–1.75)
9	Li 2017 [18]	11.0 vs. 25.0	NR	0.44 (0.24–0.80)
10	Shen 2017 [19]	8.9 vs. 15.0	31.0 vs. 36.0	0.59 (0.28–1.24)
11	Peabody 2025 [20]	9.1 vs. 12.3	15.5 vs. 18.7	0.74 (0.69–0.80)
12	Moreno 2024 [21]	6.8 vs. 17.2	19.0 vs. 23.4	0.40 (0.22–0.73)

CI, confidence interval; NPWT, negative-pressure wound therapy; NR, not reported; SSI, surgical site infection.

**Table 3:** Secondary clinical outcomes.

#	Study	Median LOS d (NPWT vs. Control)	Re-Operation %	90-Day Mortality %	Device-Related Complications %
1	López-López 2023 [10]	13 vs. 14	1.9 vs. 5.6	1.9 vs. 3.7	0
2	O’Neill 2020 [11]	8 vs. 9	5 vs. 5	0 vs. 0	0
3	Martin 2019 [12]	NR	NR	NR	0
4	Andrianello 2021 [13]	11 vs. 12	2 vs. 4	2 vs. 4	0
5	Javed 2019 [14]	9 vs. 11	3 vs. 6	0 vs. 2	1.7 (seroma)
6	Burkhart 2017 [15]	10 vs. 13	4 vs. 8	2 vs. 2	0
7	Kuncewitch 2020 [16]	9 vs. 10	4 vs. 10	0 vs. 2	0
8	Greene 2023 [17]	11 vs. 12	5 vs. 7	2 vs. 3	0
9	Li 2017 [18]	7 vs. 10	2 vs. 5	0 vs. 1	0
10	Shen 2017 [19]	10 vs. 11	4 vs. 6	1 vs. 2	0
11	Peabody 2025 [20]	NR	3.5 vs. 4.1	1.2 vs. 1.5	NR
12	Moreno 2024 [21]	9 vs. 12	3 vs. 5	0.7 vs. 1.6	0

LOS, length of stay; NPWT, negative-pressure wound therapy; NR, not reported.

**Table 4:** Summary of findings (GRADE).

Outcome (30 Days)	Participants (Studies)	Relative Effect (95% CI)	Absolute Effect	Certainty	Explanation
Superficial SSI	15,212 (12)	RR 0.71 (0.63–0.79)	126 → 90 per 1000	Moderate	Downgraded 1× for imprecision
Deep/organ-space SSI	14,456 (9)	RR 0.93 (0.79–1.09)	188 → 175 per 1000	Low	Imprecision + bias
Length of stay	9135 (10)	MD −1.7 days (−2.5 to −0.9)	–	Low	Inconsistency
Re-operation	9987 (11)	RR 0.56 (0.39–0.80)	61 → 34 per 1000	Low	Bias + imprecision
90-day mortality	12,604 (9)	RR 0.94 (0.69–1.28)	21 → 20 per 1000	Low	Imprecision
